# Synthesis and Antiviral Activity of Conformational Analogues of Leucamide A

**DOI:** 10.3390/molecules171214522

**Published:** 2012-12-07

**Authors:** Wen-Long Wang, Hai-Jun Chen, Wei-Ping Ma, Min Gu, Min-Zhi Fan, Jing-Ya Li, Bainian Feng, Fa-Jun Nan

**Affiliations:** 1 School of Pharmaceutical Science, Jiangnan University, Wuxi 214122, China; E-Mail: wenlongwang@jiangnan.edu.cn; 2 Chinese National Center for Drug Screening, Shanghai Institute of Materia Medica, Shanghai Institutes of Biological Sciences, Graduate School of Chinese Academy of Sciences, Chinese Academy of Sciences, Shanghai 201203, China; E-Mails: chj.gy@126.com (H.-J.C.); wpma@mail.shcnc.ac.cn (W.-P.M.); mgu@mail.shcnc.ac.cn (M.G.); mzfan@mail.shcnc.ac.cn (M.-Z.F.); jyli@mail.shcnc.sc.cn (J.-Y.L.)

**Keywords:** leucamide A, conformational analogues, synthesis, anti-influenza virus A

## Abstract

In order to study the effect of heterocyclic core conformational state of leucamide A on its anti-influenza virus A activity, five conformational analogues were prepared by replacing the Pro-Leu dipeptide in the molecule with various amino acids. The amino acids used were of 2 to 6 carbons. The results showed that these replacements not only changed the conformational relationship between the 4,2-bisheterocycle tandem pair and the third heterocycle, but also had dramatic effect on its activity against influenza virus A.

## 1. Introduction

**Figure 1:**
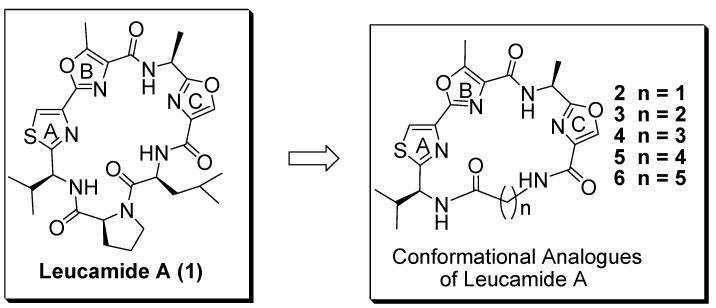
Leucamide A and its conformational analogues.

Leucamide A (**1**, Figure 1) is a bioactive cyclic heptapeptide isolated from the Australian marine sponge *Leucetta microraphis*. It contains a unique mixed 4,2-bisheterocycle tandem pair consisting of a methyloxazole and thiazole subunit. It was found to be moderately cytotoxic toward several tumor cell lines [1]. Many highly bioactive compounds also contain similar heterocyclic tandem subunits [2,3]. It was reported that the heterocyclic tandem pair plays a pivotal role in their bioactivity through specific interaction(s) with DNA or other targets [4,5,6,7,8]. One such example is the anti-tumor drug bleomycin, which interacts with DNA by binding to the nucleic bases in a mode that it is partially intercalative, penetrates positively charged moieties distal to the bithiazole into the major grove of the DNA [9]. Therefore, the unique mixed 4,2-bisheterocyclic tandem pair in leucamide A provides us with a useful scaffold for searching for compounds with therapeutic potential.

## 2. Results and Discussion

Our efforts to search for novel bioactive compounds started with leucamide A [10]. We constructed a library of 4,2-bisheterocyclic tandem derivatives consisting of a methyloxazole and thiazole subunit. Based on the bleomycin example, we hypothesized the compounds to be synthesized would have the ability to interact with DNA or RNA. Thus, the bioassay target was focused on virus in relation with DNA or RNA replication, such as influenza virus and hepatitis B virus. As we reported before, several compounds containing tandem 2,4-disubstituted methyloxazole-thiazole subunits, showed moderate activity against influenza A virus [11,12], herpes simplex type 2 virus (HSV2) [12], and hepatitis B virus (HBV) [13], while leucamide A itself did not show any antiviral activity. We postulated the conformational state of heterocyclic core might play pivotal role in their bioactivity against influenza A virus. To prove this, we synthesized a series of conformational analogues using a series of amino acids in the place of the proline-leucine dipeptide fragment in leucamide A. The difference of anti-viral activity between leucamide A and conformational analogues **2**–**6**, which have various ring sizes and different conformational states, was examined.

### 2.1. Chemistry

**Scheme 1:**
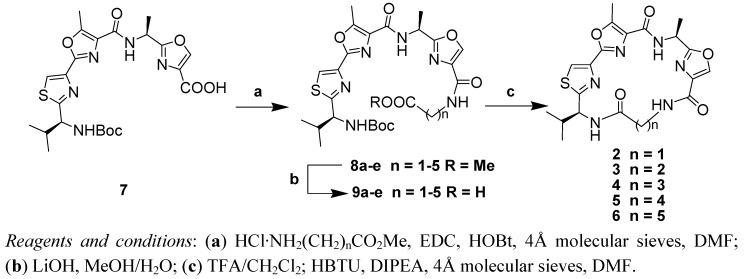
Synthesis of leucamide A conformational analogues **2**–**6**.

The analogues of leucamide A (**1**) were prepared in three steps from **7** [10] (Scheme 1). The coupling reaction of **7** with various amino acid methyl esters was accomplished using 1-(3-dimethylaminopropyl)-3-ethylcarbodiimide hydrochloride (EDC) in the presence of 1-hydroxy-benzotriazole (HOBt). This step produced the corresponding amide **8** in 73%–83% yield. The carboxylic acid group of **9** was obtained from **8** in almost quantitative yield by saponification with lithium hydroxide in aqueous methanol. The final ring-closure step was successfully implemented after deprotecting the Boc group using TFA in CH_2_Cl_2_ followed by treatment of the resulted crude amino acid with HBTU and diisopropylethylamine in dry DMF. The yields of these macrocyclization reactions varied from 25% to 49%. The macrocyclization yields were generally lower compared to that of leucamide A, which was as high as 87% [10]. Leucamide A it contains a proline residue, and proline amide bonds are known to have *cis/trans* geometry [14] and facilitate cyclization [15].

### 2.2. Biological Activities

The analogues of leucamide A were evaluated for their ability to inhibit Influenza A virus H3N2 (A3 China/15/90) replication in Madine-Darby Canine kidney (MDCK) cells [11]. The results are summarized in Table 1. The compounds **3**–**6** exhibited a general progression in relative biological activity as the size of the linker increases, especially the compound **6** [12] with an IC_50_ of 41 μM against influenza A virus, while leucamide A and compound **2** were inactive. The potency of compound **6** was comparable with that of ribavirin, which showed an IC_50_ of 16 μM. Furthermore, compounds **4**–**6** showed low cytotoxicity, which nonetheless still exhibited a reasonable selectivity index (CC_50_/IC_50_). The results indicated that the most salient difference between active and inactive analogues was their relative ability to achieve a biological conformational state.

**Table 1:** Anti-influenza A virusactivity and cytotoxicity of leucamide A and its analogues in MDCK^a^.

Compound	Leucamide A	2	3	4	5	6	Ribavirin
CC_50_^b^ (μM)	787	>273	>265	595	578	374	>8197
IC_50_^c^ (μM)	NA ^d^	NA	163	115	258	41	16

^a^ Abbreviations and strains used: MDCK, Madine–Darby canine kidney cells, influenza A H3N2 viruses (A3 China/15/90); ^b^ Concentrations that cause microscopically detectable toxicity in virus-infected cultures; ^c^ Concentrations required to reduce virus-induced CPE in MDCK cells by 50%; ^d^ No activity.

### 2.3. Conformational Analysis of the Conformational Analogues of Leucamide A

**Figure 2:**
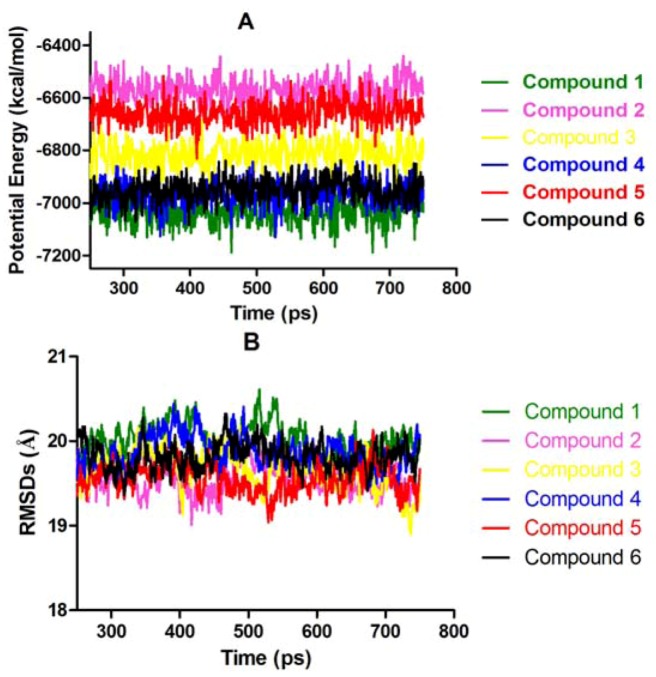
Potential energy and RMSDs profiles of compound **1**–**6** during MD simulation.

**Figure 3:**
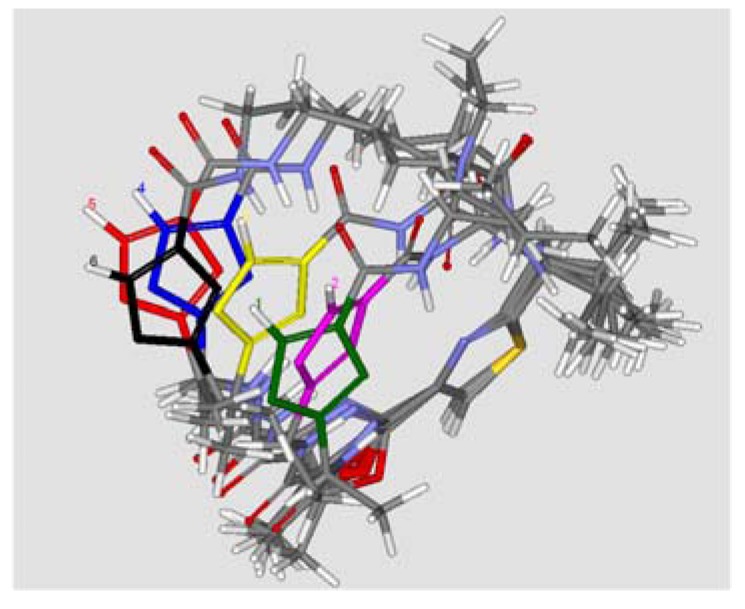
Alignment of compounds **1**–**6** and the oxazole rings (C) of compounds **1**–**6** are depicted in different colors.

To study the relationship between biological activity and the structures of leucamide A and its analogues, the molecular structures were refined by molecular dynamics (MD) simulation using the Standard Dynamics Cascade protocol of Discovery Studio 2.1 in CHARMm force field. First, these molecules were solvated in an orthorhombic solvent model with explicit periodic boundary conditions. The Standard Dynamics Cascade includes two stages of energy minimization followed by three stages of dynamics, including heating, equilibration and production. To carry out energy minimizations, the RMS gradients 0.1 kcal/mol/Å and 0.001 kcal/mol/Å were used for the steepest descent and conjugate gradient algorithms, respectively. The heating dynamics started from 50 K and gradually increased to reach the target temperature of 300 K in 50,000 steps with a time step of 1fs. Subsequently, the systems were equilibrated for 200 ps and the production dynamics were carried out for 500 ps at 300 K with the time step of 1 fs using the NPT ensemble. The conformations were obtained at every 1 ps. The root mean square deviation (RMSD) values of all the conformations were calculated by aligning all of the frames to the initial production structure. The potential energy and RMSDs of the collected snapshots of MD simulations are shown in Figure 2. The graph indicates that leucamide A and its analogues are relatively stable during the course of MD simulation. The conformations with lowest potential energy of the compounds were selected and aligned. As presented in Figure 3, the thiazole ring (A) and the oxazole ring (B) were almost coplanar in compounds **1**–**6**. As far as the third oxazole ring (C) was concerned, increasing the size of the linker in the heterocycle core gradually distorted the conformational relationship between the 4,2-bisheterocycle tandem pair and the third heterocycle. This partially supported the corresponding data and suggested that the conformation changes of heterocycle core existing in the leucamide A and its analogues, had a great influence on the bioactivity against influenza virus A.

**Figure 4:**
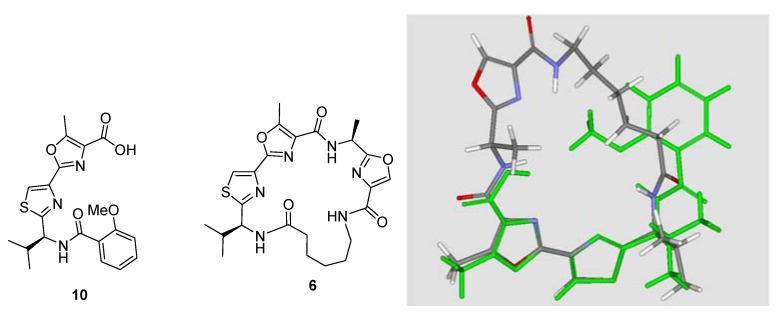
Alignment of **10** and **6**.

To further understand the structure-activity relationships, we compared the most active compound **6** with the previously reported active compound **10** [11] (with an IC_50_ value of 70 μM against influenza A virus). An overlay of **10** and **6** is shown in Figure 4. Obviously, these two compounds share a bulk hydrophobic group in a same space near the bisheterocyclic tandem pair, and are different from the other inactive compounds.

## 3. Experimental

### 3.1. Chemistry

All chemicals were reagent grade and used as purchased. All reactions were performed under an inert atmosphere of dry argon or nitrogen using distilled dry solvents. ^1^H (300 MHz) and ^13^C (75 MHz) NMR spectra were recorded on a Varian Mercury-VX300 Fourier Transform spectrometer. The chemical shifts were reported in (ppm) using the 7.26 signal of CDCl_3_ (^1^H-NMR) and the 77.23 signal of CDCl_3_ (^13^C-NMR) as internal standards. High-resolution MS data were obtained on a Finnigan MAT-95 mass spectrometer.

#### 3.1.1. General Procedure for the Preparation of Derivatives **8a**–**e**

A mixture of methyl 2-aminoacetate hydrochloride (11 mg, 0.09 mmol) and DIPEA (11 μL, 0.12 mmol) in DMF (0.5 mL) was added at −10 °C to a mixture of compound **7** (30 mg, 0.06 mmol), HOBt (16 mg, 0.12 mmol), EDC (17 mg, 0.09 mmol) and 4Å molecular sieves in DMF (1.5 mL) and the resulting mixture was stirred for 2 h with gradual warming to room temperature. The reaction mixture was diluted with EtOAc (50 mL) and H_2_O (20 mL). The aqueous phase was extracted with EtOAc. The combined organic phases were then processed in the usual way and chromatographed (1:1 petroleum ether/EtOAc) to yield compound **8a** (25 mg, 73%)*.*

*({2-[1-({2-[2-(1-tert-Butoxycarbonylamino-2-methyl-propyl)-thiazol-4-yl]-5-methyloxazole-4-carbonyl}amino)ethyl]oxazole-4-carbonyl}amino)acetic acid methyl ester* (**8a**). Yield = 73%; ^1^H-NMR (CDCl_3_): *δ* 0.90 (d, *J* = 6.9 Hz, 3H), 1.00 (d, *J* = 6.3 Hz, 3H), 1.44 (s, 9H), 1.66 (d, *J* = 6.6 Hz, 3H), 2.49 (m, 1H), 2.71 (s, 3H), 3.76 (s, 3h), 4.18 (d, *J* = 5.1 Hz, 2H), 4.49 (m, 1H), 5.26 (m, 1H), 5.45 (m, 1H), 7.45 (br, 1H), 7.57 (d, *J* = 8.1 Hz, 1H), 7.87 (s, 1H), 8.14 (s, 1H).

*3-({2-[1-({2-[2-(1-tert-Butoxycarbonylamino-2-methylpropyl)-thiazol-4-yl]-5-methyloxazole-4-carbonyl}amino)ethyl]oxazole-4-carbonyl}amino)propionic acid methyl ester* (**8b**)*.* Yield = 78%; ^1^H-NMR (CDCl_3_): *δ* 0.87 (d, *J* = 6.6 Hz, 3H), 0.96 (d, *J* = 6.6 Hz, 3H), 1.39 (s, 9H), 1.60 (d, *J* = 6.9 Hz, 3H), 2.46 (m, 1H), 2.58 (t, *J* = 6.6 Hz, 2H), 2.66 (s, 3H), 3.61 (t, *J* = 6.3 Hz, 3H), 3.64 (s, 3H), 4.90 (m, 1H), 5.34 (m, 1H), 5.41 (m, 1H), 7.41 (m, 1H), 7.56 (d, *J* = 8.7 Hz, 1H), 7.84 (s, 1H), 8.08 (s, 1H); ^13^C-NMR (75 MHz, CDCl_3_) *δ* 11.70, 17.02, 19.15, 19.31, 28.14, 33.06, 33.79, 34.36, 42.56, 51.66, 58.04, 80.04, 119.86, 129.48, 135.87, 141.12, 142.91, 153.74, 154.22, 155.29, 160.35, 160.95,163.90, 172.36, 175.06.

*4-({2-[1-({2-[2-(1-tert-Butoxycarbonylamino-2-methylpropyl)thiazol-4-yl]-5-methyloxazole-4-carbonyl}amino)ethyl]oxazole-4-carbonyl}amino)butyric acid methyl ester* (**8c**). Yield = 80%; ^1^H-NMR (CDCl_3_): *δ* 0.87 (d, *J* = 6.9 Hz, 3H), 0.96 (d, *J* = 6.6 Hz, 3H), 1.40 (s, 9H), 1.60 (d, *J* = 7.2 Hz, 3H), 1.87 (m, 2H), 2.32 (t, *J* = 7.5 Hz, 2H), 2.44 (m, 1H), 2.66 (s, 3H), 3.41 (m, 2H), 3.61 (s, 3H), 4.91 (m, 1H), 5.30 (m, 1H), 5.40 (m, 1H), 7.11 (br, m), 7.54 (d, *J* = 8.4 Hz, 1H), 7.82 (s, 1H), 8.08 (s, 1H).

*5-({2-[1-({2-[2-(1-tert-Butoxycarbonylamino-2-methylpropyl)thiazol-4-yl]-5-methyloxazole-4-carbonyl}amino)ethyl]oxazole-4-carbonyl}amino)pentanoic acid methyl ester* (**8d**). Yield = 81%; ^1^H-NMR (CDCl_3_): *δ* 0.86 (d, *J* = 6.9 Hz, 3H), 0.95 (d, *J* = 6.9 Hz, 3H), 1.39 (s, 9H), 1.59 (d, *J* = 6.9 Hz, 3H), 1.55–1.65 (m, 4H), 2.29 (t, *J* = 6.9 Hz, 2H), 2.43 (m, 1H), 2.65 (s, 3H), 3.51 (m, 2H), 3.59 (s, 3H), 4.90 (m, 1H), 5.33 (d, *J* = 8.7 Hz, 1H), 5.39 (m, 1H), 7.09 (br, 1H), 7.57 (d, *J* = 8.4 Hz, 1H), 7.81 (s, 1H), 8.08 (s, 1H).

*6-({2-[1-({2-[2-(1-tert-Butoxycarbonylamino-2-methylpropyl)thiazol-4-yl]-5-methyloxazole-4-carbonyl}amino)ethyl]oxazole-4-carbonyl}amino)hexanoic acid methyl ester* (**8e**). Yield = 83%; ^1^H-NMR (CDCl_3_): *δ* 0.90 (d, *J* = 6.9 Hz, 3H), 0.99 (d, *J* = 6.6 Hz, 3H), 1.43 (s, 9H), 1.59–1.69 (m, 6H), 1.63 (d, *J* = 7.8 Hz, 3H), 2.29 (t, *J* = 7.5 Hz, 2H), 2.48 (m, 1H), 2.70 (s, 3H), 3.37 (m, 2H), 3.63 (s, 3H), 4.95 (m, 1H), 5.27 (m, 1H), 5.44 (m, 1H), 7.01(m, 1H), 7.53 (d, *J* = 8.7 Hz, 1H), 7.84 (s, 1H), 8.10 (s, 1H); ^13^C-NMR (75 MHz, CDCl_3_): *δ* 11.81, 17.04, 19.29, 19.41, 24.50, 26.35, 28.24, 29.29, 33.16, 33.82, 38.74, 42.62, 51.41, 58.12, 80.24, 119.86, 129.58, 136.19, 141.00, 143.08, 154.11, 154.33, 155.35, 160.34, 161.04, 163.83, 173.92, 175.28.

#### 3.1.2. General Procedure for the Preparation of Derivatives **2**–**6**

Lithium hydroxide monohydrate (11 mg, 0.25 mmol) was added to a stirred solution of **8a** (25 mg, 0.042 mmol) in MeOH/H_2_O (0.75 mL/0.25 mL) at 0 °C and stirred for 1 h with gradual warming to room temperature. TLC monitoring showed complete consumption of starting material. The solvents were removed and the residue was partitioned between EtOAc (10 mL) and H_2_O (5 mL). The organic phase was separated and the aqueous phase was acidified to pH = 2 with aqueous HCl (1 M) and then extracted with EtOAc. The combined organic phases were then processed in the usual way to afford **9a** (24 mg). To a stirred solution of **9a** (24 mg, 0.042 mmol) in CH_2_Cl_2_ (0.7 mL) at –10 °C was added TFA (0.7 mL) dropwise. The reaction mixture was stirred for 4 h with gradual warming to room temperature. TLC monitoring showed complete consumption of starting material. The solvents were removed and followed by evaporation to dryness by azeotropic distillation with toluene. A suspension of the crude solid residue and 4 Å molecular sieves in dry DMF (10 mL) was cooled to −10 °C, and diisopropylethylamine (15 μL, 0.084 mmol) and HBTU (42.5 mg, 0.112 mmol) were added. The resulting mixture was stirred at −10 °C for 2 h and then at room temperature for 2 days, the mixture was diluted with EtOAc (100 mL) and H_2_O (20 mL). The organic phases were then processed in the usual way and chromatographed (1:4 petroleum ether/EtOAc) to afforded compound **2** (4.7 mg, 25%) as a white solid.

*Compound* **2**. Yield = 25%; ^1^H-NMR (300 MHz, CDCl_3_): *δ* 0.96 (d, *J* = 6.6 Hz, 3H), 1.05 (d, *J* = 6.3 Hz, 3H), 1.74 (d, *J* = 6.6 Hz, 3H), 2.25 (m, 1H), 2.70 (s, 3H), 3.87 (d, *J* = 16.8 Hz, 1H), 4.78 (dd, *J* = 17.1 Hz, *J* = 8.4 Hz, 1H), 5.08 (m, 1H), 5.47 (m, 1H), 7.52 (d, *J* = 8.7 Hz, 1H), 7.59 (d, *J* = 7.2 Hz, 1H), 7.79 (s, 1H), 8.27 (s, 1H), 9.32 (d, *J* = 4.5 Hz, 1H); ^13^C-NMR (75 MHz, CDCl_3_): *δ* 11.55, 17.50, 19.06, 19.21, 34.88, 43.87, 45.23, 56.56, 119.18, 130.29, 135.23, 141.04, 142.75, 152.13, 153.85, 160.91, 160.98, 165.34, 167.94, 170.60; HREIMS *m/z* 458.1399 (calcd for C_20_H_22_N_6_O_5_S 458.1420).

*Compound* **3***.* Yield = 25%; ^1^H-NMR (300 MHz, CDCl_3_): *δ* 0.95 (d, *J* = 6.6 Hz, 3H), 1.12 (d, *J* = 6.6 Hz, 3H), 1.67 (d, *J* = 6.9 Hz, 3H), 2.40 (m, 1H), 2.60 (m, 1H), 2.72 (s, 3H), 2.80 (m, 1H), 3.73 (m, 1H), 4.10 (m, 1H), 5.11 (m, 1H), 5.49 (m, 1H), 6.36 (br, 1H), 7.87 (s, 1H), 8.08 (br, 1H), 8.19 (s, 1H), 8.70 (d, *J* = 5.1 Hz, 1H); ^13^C-NMR (75 MHz, CDCl_3_): *δ* 11.60, 16.39, 19.77, 20.26, 34.00, 34.16, 35.91, 44.68, 56.60, 120.26, 129.96, 135.90, 142.08, 142.18, 152.86, 153.91, 160.07, 160.86, 164.62, 171.70, 173.20; HREIMS *m/z* 472.1533 (calcd for C_21_H_24_N_6_O_5_S 472.1576).

*Compound* **4**. Yield = 31%; ^1^H-NMR (300 MHz, CDCl_3_): *δ* 0.91 (d, *J* = 6.6 Hz, 3H), 1.04 (d, *J* = 6.9 Hz, 3H), 1.67 (d, *J* = 6.6 Hz, 3H), 2.00–2.10 (m, 2H), 2.26 (m, 1H), 2.48–2.71 (m, 2H), 2.74 (s, 3H), 3.39 (m, 1H), 3.56 (m, 1H), 5.29 (m, 1H), 5.45 (dd, *J* = 9.3 Hz, 3.9 Hz, 1H), 7.22 (br, 1H), 7.45 (br, 1H), 7.87 (s, 1H), 8.20 (s, 1H), 8.27 (d, *J* = 7.2 Hz, 1H); ^13^C-NMR (75 MHz, CDCl_3_) δ 11.69, 16.94, 19.10, 20.61, 24.69, 33.58, 35.18, 38.64, 44.23, 56.15, 120.25, 129.65, 135.98, 140.91, 141.96, 153.52, 154.33, 160.43, 160.63, 164.59, 171.97, 172.41; HREIMS *m/z* 486.1680 (calcd for C_22_H_26_N_6_O_5_S 486.1685).

*Compound* **5**. Yield = 41%; ^1^H-NMR (300 MHz, CDCl_3_): *δ* 0.98 (d, *J* = 6.6 Hz, 6H), 1.64 (d, *J* = 6.9 Hz, 3H), 1.90 (m, 2H), 2.19–2.26 (m, 1H), 2.30–2.45 (m, 4H), 2.74 (s, 3H), 3.46 (m, 1H), 3.59 (m, 1H), 5.35–5.40 (m, 2H), 7.08 (d, *J* = 9.0 Hz, 1H), 7.36 (br, 1H), 7.86 (s, 1H), 8.09 (d, *J* = 7.8 Hz, 1H), 8.20 (s, 1H); ^13^C-NMR (75 MHz, CDCl_3_): *δ* 11.73, 18.06, 19.16, 20.83, 23.51, 29.38, 34.90, 37.02, 37.48, 43.73, 55.90, 120.07, 129.62, 136.12, 141.52, 141.88, 153.75, 154.06, 160.03, 160.56, 164.59, 172.07, 172.55; HREIMS *m/z* 500.1854 (calcd for C_23_H_28_N_6_O_5_S 500.1842).

*Compound* **6***.* Yield = 49%; ^1^H-NMR (300 MHz, CDCl_3_): *δ* 0.93 (d, *J* = 6.9 Hz, 3H), 1.00 (d, *J* = 6.6 Hz, 3H), 1.42–1.47 (m, 2H), 1.50–1.78 (m, 4H), 1.64 (d, *J* = 6.6 Hz, 3H), 2.21–2.33 (m, 2H), 2.43 (m, 1H), 2.74 (s, 3H), 3.24 (m, 1H), 3.57 (m, 1H), 5.38–5.45 (m, 2H), 7.00 (d, *J* = 8.7 Hz, 1H), 7.25 (br, 1H), 7.89 (s, 1H), 8.01 (d, *J* = 8.1 Hz, 1H), 8.16 (s, 1H); ^13^C-NMR (75 MHz, CDCl_3_): *δ* 11.79, 17.63, 19.13, 20.64, 24.60, 25.31, 27.94, 35.30, 36.63, 37.62, 43.58, 55.93, 120.58, 129.72, 136.09, 141.38, 141.60, 153.98, 154.22, 160.11, 160.66, 164.34, 172.06, 172.94; HREIMS *m/z* 514.2010 (calcd for C_24_H_30_N_6_O_5_S 514.1998).

### 3.2. Biological Assays [11]

MDCK cells were grown as specified in Eagle’s minimum essential medium with 10% heat-inactivated fetal bovine serum (FBS) plus antibiotics (penicillin, 100 U/mL; streptomycin, 100 U/mL). Influenza A H3N2 viruses (A3 China/15/90) were propagated in the allantoic cavities of 10-day old embryonated eggs. Virus titers were determined by hemagglutinin titration, according to standard procedures. Confluent MDCK monolayers were infected with influenza A viruses for 2 h at 37 °C, after which the viral inoculum was removed and cells were treated with different concentrations of compound. When CPE result of the viral control group reached 4+, the result of compound treated group was observed. The dilution that gives 50% cytopathic effect was determined by the interpolating procedure of Reed and Muench.

## 4. Conclusions

In summary, we have prepared five conformational analogues of leucamide A, by replacing the Pro-Leu dipeptide of leucamide A with various amino acids. The compounds **3**–**6** showed activity against influenza virus A. The inability of leucamide A and compound **2** to exhibit comparable biological action underscores the importance of the topological conformation requirements for bisheterocyclic tandem pairs to interact with biological targets. From these results, combined with the results we reported previousely [11], the conclusion is that the bisheterocyclic tandem pairs with a third heterocyclic or even the bisheterocyclic tandem pairs alone present a useful scaffold for searching for novel antiviral compounds, and the introduction of a bulk hydrophobic group in certain positions might lead to enhancement of antiviral potency. Further efforts focused on the simple open chain bisheterocyclic tandem derivatives are in progress.
